# Malignant Bone Tumors Diagnosis Using Magnetic Resonance Imaging Based on Deep Learning Algorithms

**DOI:** 10.3390/medicina58050636

**Published:** 2022-05-04

**Authors:** Vlad Alexandru Georgeanu, Mădălin Mămuleanu, Sorin Ghiea, Dan Selișteanu

**Affiliations:** 1 Department of General Medicine, “Carol Davila” University of Medicine and Pharmacy, 050474 Bucharest, Romania; vgeorgeanu@hotmail.com; 2 Orthopaedics and Trauma Surgery Department, “St. Pantelimon” Hospital, 021659 Bucharest, Romania; 3 Department of Automatic Control and Electronics, University of Craiova, 200585 Craiova, Romania; dan.selisteanu@edu.ucv.ro; 4 Oncometrics S.R.L., 200677 Craiova, Romania; 5 Monza Hospital, 021967 Bucharest, Romania; sorin.ghiea@gmail.com

**Keywords:** bone tumors, deep learning, convolutional neural networks

## Abstract

*Background and Objectives*: Malignant bone tumors represent a major problem due to their aggressiveness and low survival rate. One of the determining factors for improving vital and functional prognosis is the shortening of the time between the onset of symptoms and the moment when treatment starts. The objective of the study is to predict the malignancy of a bone tumor from magnetic resonance imaging (MRI) using deep learning algorithms. *Materials and Methods*: The cohort contained 23 patients in the study (14 women and 9 men with ages between 15 and 80). Two pretrained ResNet50 image classifiers are used to classify T1 and T2 weighted MRI scans. To predict the malignancy of a tumor, a clinical model is used. The model is a feed forward neural network whose inputs are patient clinical data and the output values of T1 and T2 classifiers. *Results*: For the training step, the accuracies of 93.67% for the T1 classifier and 86.67% for the T2 classifier were obtained. In validation, both classifiers obtained 95.00% accuracy. The clinical model had an accuracy of 80.84% for training phase and 80.56% for validation. The receiver operating characteristic curve (ROC) of the clinical model shows that the algorithm can perform class separation. *Conclusions*: The proposed method is based on pretrained deep learning classifiers which do not require a manual segmentation of the MRI images. These algorithms can be used to predict the malignancy of a tumor and on the other hand can shorten the time of their diagnosis and treatment process. While the proposed method requires minimal intervention from an imagist, it needs to be tested on a larger cohort of patients.

## 1. Introduction

In the last period, a significant increase in the number of bone tumors in general and malignant tumors in particular was noted. Although the percentage of bone malignant tumors is quite low compared to benign ones, they are a major problem due to their aggressiveness and relatively low survival rate, regardless of the method of treatment applied. One of the determining factors for improving vital and functional prognosis is the speed with which the diagnosis is made.

The diagnosis of bone tumors is based on three elements: symptomatology, imaging, and histopathologic aspect. Unfortunately, the symptoms are completely non-specific, and they provide little data to guide the diagnosis to one of the two major categories of bone tumors, especially in the early stages of the disease. The histopathologic examination is the defining element, which provides the most accurate diagnosis and establishes the therapeutic approach. Given its invasive nature, this examination represents the final stage of diagnostic algorithm. For maximum accuracy, imaging data are necessary to determine the most appropriate harvesting area. Due to recent major technological advances, imaging investigations have become increasingly successful. Currently there are many methods used in the study of bone tumors including classical radiology, computed tomography (CT), magnetic resonance imaging (MRI), angiography, scintigraphy, and positron emission tomography—computed tomography (PET-CT). 

This paper proposes a method of predicting the malignancy of bone tumors based on MRI. The dataset contains MRI scans with diagnoses confirmed by histopathological investigations. Two pretrained residual convolutional neural networks are used (CNNs) to classify the images extracted from the MRI dataset, for T1 and T2. By using pretrained CNNs, the model is converging fast during training, and it can train on a small dataset. While other studies have proposed a manual segmentation of the images [1], in our study the images are not manually segmented but are sent to the training pipeline as a whole in order to predict the malignancy based on the entire image. In addition, the proposed clinical model has as inputs the values predicted by the two residual CNNs models and the bone location of the tumor.

## 2. Related Work

At this moment, there are many studies which are analyzing how deep learning and machine learning algorithms can have a positive impact in the healthcare field. Giovanni Briganti et al. [2] reviewed the development of AI in clinical practice in fields such as cardiology, pulmonary, endocrinology, neurology, concluding that it is a very promising approach. In other fields of biomedicine, such as mammalian cell cultures, algorithms are developed to overcome specific issues [3]. Bobak J. Mortazavi et al. [4] concluded that ML algorithms can improve the prediction of heart stroke compared with classical statistical methods. With the significant progress of CNNs in feature extraction or classification, several architectures were developed with specific applications for medical imaging, such as image segmentation [5,6], automatic detection of lesions [7], 3D volumetric segmentation [8] or image reconstruction using generative adversarial networks (GAN) [9]. AI algorithms can be effective preoperative and are used to detect, classify, or segment bone lesions in medical imaging. Assessment of bone and soft-tissue sarcoma’s therapeutic response is achieved traditionally by one-dimensional or 3-D measurements (Response Evaluation Criteria in Solid Tumors (RECIST)). Multiparametric or functional MRI which includes diffusion-weighted imaging (DWI) and dynamic contrast-enhanced (DCE) MRI is another efficient technique for assessment of treatment response and evaluation of postsurgical residual or recurrent disease. The AI algorithms can be effective for postoperative evaluation as well [10,11].

In the field of orthopedics, there are quite few studies related to the use of AI techniques in the classification and characterization of bone tumors. Yu He et al. [12] proposed a deep learning model for bone lesion classification on radiographs. The dataset used in their study had 2899 images and the lesions were histologically confirmed. The algorithm used was an EfficientNet-B0 convolutional model pretrained on ImageNet dataset and the model performance was evaluated using receiver operating characteristic (ROC) curves and area under curve (AUC). In their study, Yu He et al. [12] obtained values for AUC between 0.894 and 0.916. Other studies used magnetic resonance imaging and deep learning models for classifying different bone lesions and obtained good performance on the observed metrics. Keyang Zhao et al. [13] proposed a diagnosis method of musculoskeletal tumors based on DL algorithms. Their proposed method used contrast enhanced MRI scans. Their method shows that software based on DL algorithms can significantly improve the diagnosis given by an orthopedist. Salvatore Gitto et al. [14] proposed an algorithm for classification of atypical cartilaginous tumor and grade II chondrosarcoma of long bones. The inputs of their study were 93 MRI scans on which several procedures were applied until the training of the machine learning model such as segmentation, filtering, feature extraction, feature selection, and oversampling. The machine learning model method they proposed is highly accurate in distinguishing atypical cartilaginous tumor from grade II chondrosarcoma. In terms of image segmentation, Jia Wu et al. [15] proposed a method based on convolutional neural networks with image preprocessing and tumor area calculation for segmentation of osteosarcoma in MRI scans. In our previous work [16], a CNN approach along with image processing techniques were used to classify bone MRI scans, and the performance of two pretrained CNN models was also analyzed and compared.

## 3. Materials and Methods

**Figure 1:**
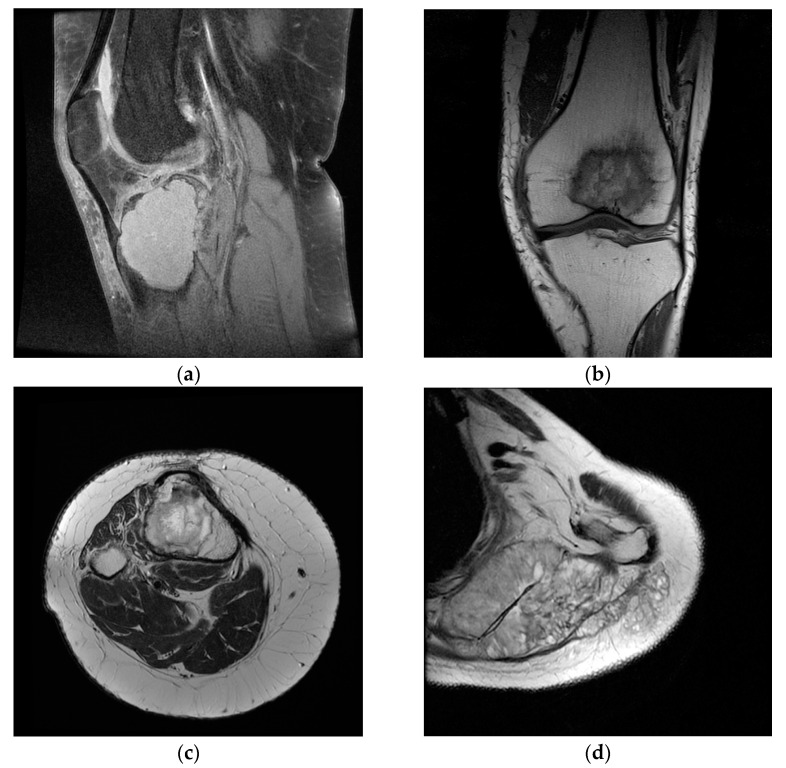
Sample images from the dataset used in the study. (**a**) Benign tumor T1 FS weighted image; (**b**) malignant tumor T1 weighted image; (**c**) benign tumor T2 weighted image; (**d**) malignant tumor T2 weighted image.

MRI provides qualitative information regarding tumor contents such as fat, water, and blood on the basis of the signal patterns. Regions with low signal intensity on T1-weighted images and high signal intensity on T2-weighted images reflect free water in the lesion. Regions of high intensity on both the T1-and T2-weighted images reflect fat. Regions of low intensity on both the T1-and T2-weighted images reflect cortical bone, fibrotic tissue, calcifications, or air. Contrast-enhanced MRI can appreciate the vascularization of the tumor and the evolution of this aspect after neoadjuvant therapy. In this way, the MRI guidance makes it possible to avoid biopsying necrotic areas. MRI is very helpful in local staging and surgical planning by assessing the degree of intramedullary extension and invasion of the adjacent physeal plates, joints, muscle compartments and neuro-vascular bundles. It can be used in assessing response to neoadjuvant therapy and further restaging. The post-therapeutic follow-up should also be carried out using MRI. The T1 signal intensity is compared to that of the muscle. This sequence is very important in the evaluation of bone marrow. Most bone tumors will be identified as lesions with low signal against a background of surrounding fatty marrow [17]. In order to improve the tumor’s vascularization aspect, suppressing the fat signal in T1 after injection of gadolinium can be useful. This contrast-enhanced MRI (CEMRI) can be useful in the differentiation between solid (hyperenhancing) and fluid-containing (non-enhancing) lesions. The reference for T2-weighted imaging is fat, particularly in anatomic regions where there is relatively little muscle. The bone tumors with a significant chondroid/cartilaginous or liquid component have a high signal intensity on T2 [18]. Water shows higher signal than fat on T2; suppressing the fat signal on this sequence can evaluate the presence and the extent of oedema, a very important element of bone tumors. CEMRI with gadolinium can improve the distinction of oedema from viable tumors. The tumor-associated oedema often does not correlate with the degree of malignancy or tumor aggressiveness. The distinction between tumor and oedema can be difficult but is essential for extension tumor appreciation. Tumoral tissue has a more heterogeneous signal than that of associated oedema [19]. The local extension of the tumor is another very important aspect well appreciated by MRI. Direct extension of the tumors into the articular cavity through a destroyed cortex is a direct sign of joint involvement, as opposed to joint effusion. Muscular invasion should be assessed by T1 and T2 with fat suppression as well. MRI, especially T2 with fat suppression, is the best method for the evaluation of neurovascular involvement, even better than CT or angiography. The most specific aspects are loss of the perivascular/perineural fat and associated stenosis [17]. The MRI is useful for the assessment of the evolution of the tumor after neo-adjuvant therapy or radiotherapy. Reduction of tumor volume, decrease of associated oedema and extent of marrow invasion are favorable indicators. The amount of necrosis is another predictive indicator. It is considered good (≥90% tumors necrosis) or poor (<90% tumors necrosis) [20]. Unfortunately, it is hard to be appreciated by direct observation; different types of software would likely become more useful in this direction [17]. Despite the fact that most bone tumors are resistant to radiotherapy, expected post-radiation MRI changes are the decrease of the tumors size and an increase in its T2 signal intensity (due to fatty transformation of the bone marrow) [21]. Distinction between recurrences and post therapeutic changes is another very sensitive topic which can be solved by MRI. Marrow replacement and cortical disruption are defining imaging elements for tumor recurrence. Lesions with low signal intensity on T2 generally do not represent recurrent tumors (sensitivity 96%), in contrast to high signal lesions [22]. Post-therapeutic changes (vascularized granulation tissue, neovascularization) usually lack high signal intensity on T2. The dataset used in our study contains 39 magnetic resonance image scans (MRI) with malignant and benign tumors confirmed by histopathological investigations. This research incorporates patients both men and women, who reached the ages of 15 to 80 years old between 2019 and 2021. Lesion location, bone type, patient age and gender are presented in Table 1. For each MRI scan in the dataset, the images for all three planes were extracted (i.e., sagittal, coronal, and transverse plane for both T1 weighted and T2 weighted images). The goal of this preprocessing step was to create two types of datasets from the MRI scans: T1 weighted images containing malignant and benign tumors and T2 weighted images containing malignant and benign tumors. After this step, a total of 1458 T1 weighted images were exported containing malignant and benign tumors and 648 T2 weighted images containing also malignant and benign tumors. Before the preprocessing step, each image was reviewed by a radiologist to detect anomalies in the images exported. Sample image tiles are presented in Figure 1.

**Figure 2:**
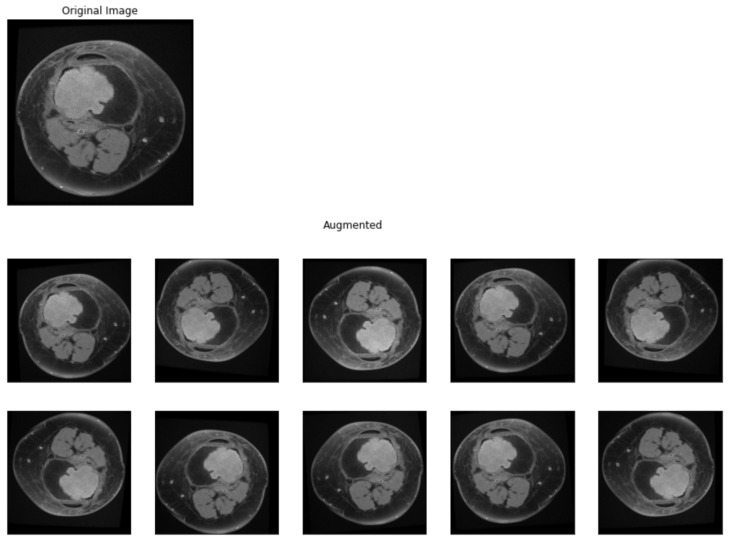
Data augmentation. Original sample and augmented samples.

Only images containing lesions were selected from the MRI scans, exported from DICOM (Digital Imaging and Communications in Medicine) format and labeled according to the type of lesion, and placed in different folders (0 for benign tumors, respectively, and 1 for malignant tumors). In order to obtain a model with significant performance, a large and curated dataset is needed. For the dataset used in the study, different image processing techniques were applied, with the main goal of having enough data for training a model. Data augmentation is a preprocessing technique in which sample from the dataset is chosen and, by applying different operations on it, other samples are obtained, similar with the original one but not different enough to change the label of the input. Data augmentation techniques can prevent a deep learning model to overfit the training data [23]. To generate new data, the following operations were applied for each image in the dataset: brightness change, contrast change, horizontal flip, vertical flip, rotation (0–15 degrees), width shift, height shift, and shear. The augmented images obtained after this process are presented in Figure 2. After this step, all images were resized to a 224 by 224 by 3 tensor using cubic interpolation.

## 4. Proposed Method

**Figure 3:**
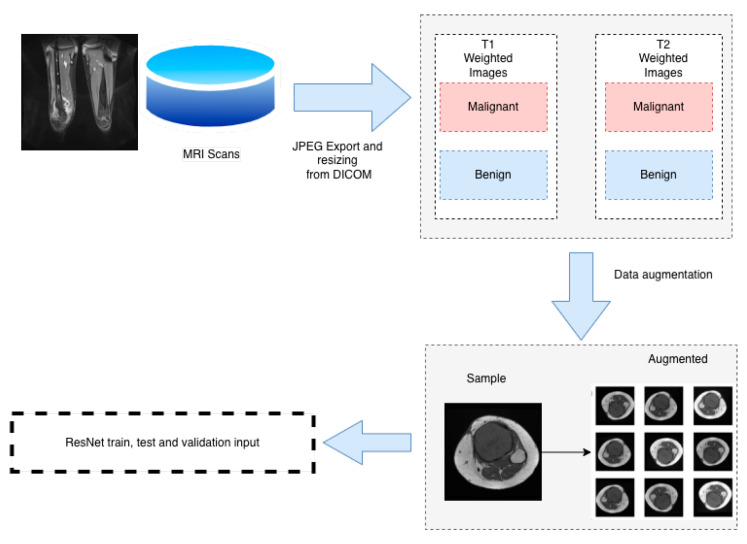
Dataset pipeline.

**Figure 4:**
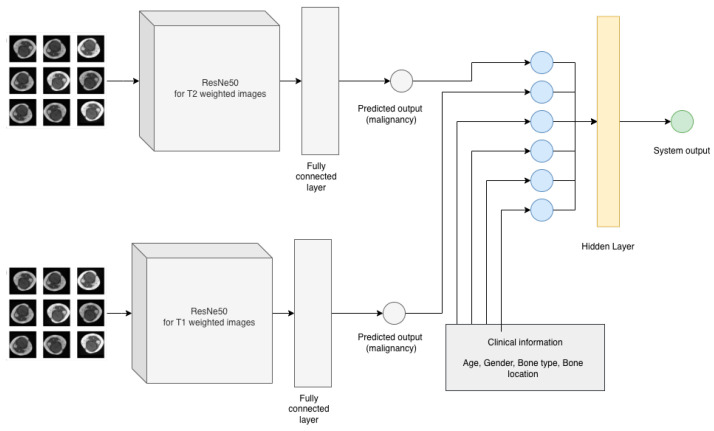
Proposed methodology.

The goal of the study is to create a model that can predict the malignancy of a bone tumor based on MRI scan and clinical data of the patient. In order to address this, the challenge was divided into three components. The diagram of the proposed methodology is presented in Figure 3 and Figure 4. The first two components of the proposed system contain two residual networks models. The model used for each component is ResNet50, which is a CNN model proposed by K. He et al. [24] and has 50 convolutional layers in depth. K. He et al. [24] introduced residual blocks to address the vanishing gradients problem [25]. The authors proposed the layers of a CNN model to fit a residual mapping instead of stacking each layer directly. Since it was introduced in 2015, ResNet was used in many classification tasks [26,27]. In healthcare, ResNet has been used with success in tasks such as pneumonia detection in chest Xray images [28], knee anterior cruciate ligament detection [29], breast cancer detection [30]. The ResNet50 [24] architecture of the proposed system can be found in Table 2. Feyisope R. et al. [1] proposed a similar methodology for classifying bone tumors in which they have two models for predicting the output and a logistic regression for the clinical model. In their study, the MRI scans were manually segmented by a radiologist and the resulting images were used for training. Also, for the clinical model, Feyisope R. et al. [1] used as input the following information: sex, age, and lesion location. The image classifier used in their study is a pretrained EfficientNet [31]. ResNet50 is a deep learning model which needs a very large dataset to train. For the proposed methodology, each ResNet50 model was trained using transfer learning techniques. The weights loaded into each model were obtained from training each network on the ImageNet dataset. The dataset contains around 14 million labeled images with 20,000 classes [32]. Since the purpose of each pretrained ResNet50 model is to predict the malignancy from the MRI scan, the last fully connected layer was dropped and a layer containing one neuron was added.

Since each ResNet50 model used in the proposed methodology has to predict the malignancy of a given tumor, the activation function for the last layer is Sigmoid and is given by Equation (1). The loss function used for the classifiers is binary cross entropy (*BCE*) and, if *N* represents the total number of samples, *y_i_* label for sample *i* and *p*(*y_i_*) is the predicted probability, it can be expressed as Equation (2). The optimizer chosen is RMSprop [33] with a learning rate of 0.0001.

(1) $$y=\frac{1}{1+e^{-x}}$$

(2) $$BCE=-\frac{1}{N}\;\overset{N}{\underset{i=1}{\sum }}y_{i}\cdot \log\left(p\left(y_{i}\right)\right)+\left(1-y_{i}\right)\cdot log\left(1-p\left(y_{i}\right)\right)$$

The dataset was randomly split in 70% for training, 20% for testing and 10% for validation and both ResNet50 models were trained for 30 epochs with a batch size of 20 and 15 steps per epoch and 5 validation steps.

**Figure 5:**
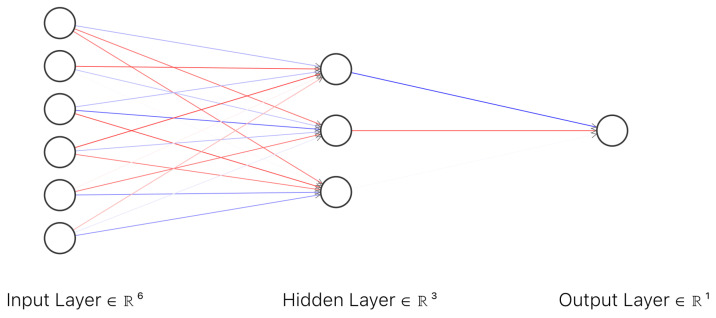
Architecture of the artificial neural network for the clinical model.

In order to prepare the dataset for the clinical model the demographic information about the patients in the study (gender, bone type and lesion location) were numerically encoded as described in Table 3. As an additional input to the clinical model, at least two random images (T1 weighted image and T2 weighted image) were selected for each MRI scan. These images were selected from the dataset built for ResNet50 classifiers, dataset which contained only images with lesions (malignant or benign). The images were introduced to the corresponding ResNet50 classifier in order to obtain the predicted malignancy for each selected image. The next step was to compute the average of the predicted malignancy for each ResNet model and add the obtained values to the clinical model dataset as T1 predicted output and T2 predicted output. Eight MRI scans did not contain T2 imaging and hence, the dataset for the clinical model had missing values for column T2 predicted malignancy. For these missing values, the standard deviation of predicted values for T2 was used with the aim of not affecting the final output. Assuming that $\bar{x}$ is the arithmetic mean of the observations for column T2 predicted malignancy, standard deviation can be expressed as Equation (4). The clinical model is an artificial neural network with an input layer of six neurons, one hidden layer with three neurons and one neuron as an output (Figure 5). The activation function for the first two layers is rectified linear unit (ReLu) and can be expressed by Equation (3) and the activation function for the output layer is Sigmoid (1).

(3) $$\sigma \left(x\right)=\{\begin{array}{c} \max\left(0,x\right),\;x\geq 0\\ 0,\;x<0 \end{array}$$

(4) $$Std\;Dev=\sqrt{\frac{1}{N}\;\overset{N}{\underset{i=1}{\sum }}{\left(x_{i}-\bar{x}\right)}^{2}}$$

The loss function for the clinical model is binary cross entropy (2) and the optimizer is RMSProp [33] with a learning rate of 0.001. Before training the clinical model, oversampling techniques were applied to the dataset. All of the samples from the clinical model dataset were duplicated with the purpose of assuring adequate samples for training and validation of the clinical model. After this step, 241 samples were obtained, and this dataset was randomly split into 80% samples for training and 20% samples for validation. The model was trained for 50 epochs with a batch size of 120.

## 5. Results

The dataset used in the proposed methodology contains 39 MRI with both malignant and benign tumors confirmed by histopathological investigations. The MRI scans are obtained from 23 patients with ages between 15 and 80, both women and men. For measuring the performance of the image classifiers (ResNet50) models, accuracy (5), precision (6), recall (7), and area under the curve have been measured. The performance of the T1 and T2 weighted images classifiers is presented in Table 4. The values are separated from training and validation steps and the chosen threshold is 0.6. On the validation step, the image classifier for T1 weighted images achieved an accuracy of 97.00%, recall of 95.65% and area under the curve of 1.00. The T2 weighted image classifier for the validation step, obtained an accuracy of 95.00%, recall of 95.52% and area under the curve of 0.9923.

(5) $$Accuracy=\frac{TP+TN}{TP+TN+FP+FN}$$

(6) $$Precision=\frac{TP}{TP+FP}$$

(7) $$Recall=\frac{TP}{TP+FN}$$

Similarly, accuracy, recall, area under the curve, and receiver operating characteristic curve (ROC curve) were observed for the clinical model. The obtained values for the clinical model are presented in Table 5. 

**Figure 6:**
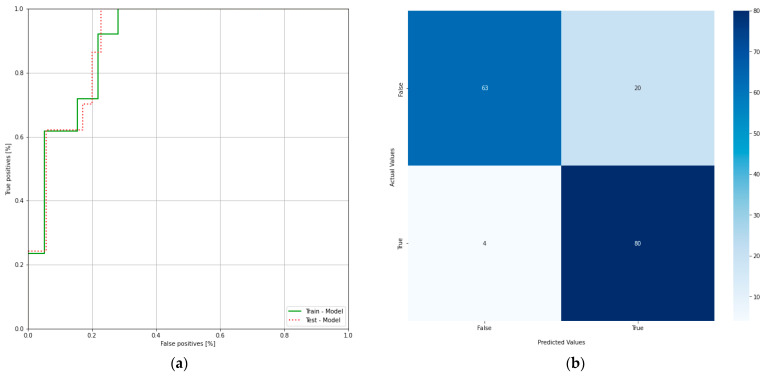
(**a**) Receiver operating characteristic curve of the clinical model; (**b**) confusion matrix of the clinical model.

ROC curve illustrates the performance of a binary classifier and it is obtained by plotting true positives and false positives at different threshold settings and the confusion matrix of the proposed system (Figure 6a,b). The area under the ROC curve of a binary classifier is 0.5. From the ROC curve, it can be concluded that the obtained model is performing well at different thresholds on the data used in the study.

## 6. Discussion

**Figure 7:**
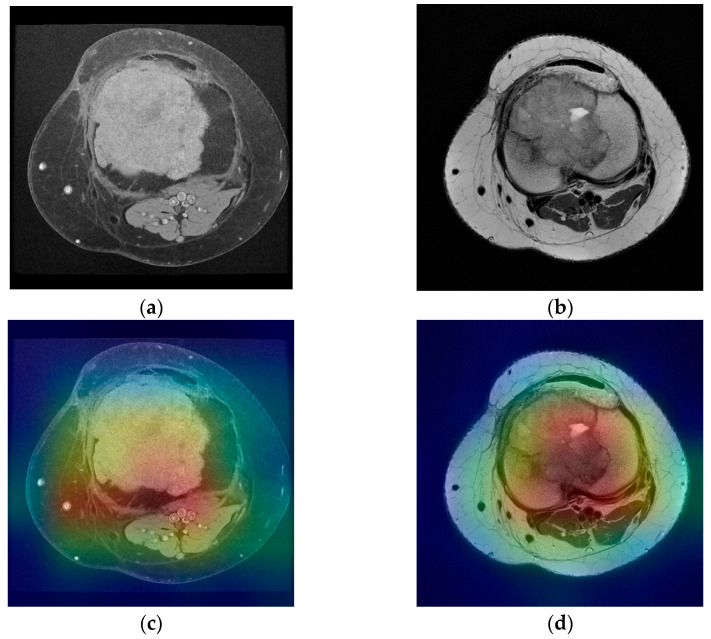
(**a**) Tibia benign tumor -T1 FS weighted image; (**b**) tibia benign tumor—T2 weighted image; (**c**,**d**) corresponding class activation maps.

The first stage of imaging diagnosis is classical radiology. This determines the location of the tumor inside or on the surface of the bone and position related to anatomical regions (epiphysis, metaphysis, diaphysis). It also establishes the nature of the tumor (osteolytic, osteocondensing or mixed), the boundary between it and the surrounding bone, as well as the presence of the periosteal reaction. All of these elements can guide the diagnosis to a certain type of bone tumor; only in a few cases, the elements are so clear that a definite diagnosis can be made. CT is a useful diagnostic method, providing essential data on tumor matrix mineralization, calcification, ossification, cortical scalloping, or destruction and periosteal reaction. CT images with high resolution can detect even small lesions (larger than 3 mm). Regarding the diagnosis of metastases, CT of the chest, abdomen, and pelvis is the main method of highlighting them (as well as regional lymphadenopathy). The biopsy under CT guidance is particularly precise, currently representing the gold standard. MRI provides qualitative information regarding tumor contents such as fat, water, and blood on the basis of the signal patterns. Malignant tumors are commonly associated with sizes larger than 8 cm, irregular margins, inhomogeneous signals, and the presence of edema, necrosis, hemorrhage, fascial penetration, bone changes and neurovascular involvement. The highest sensitivity for malignancy prediction is high signal intensity on T2-weighted images, heterogeneous signal intensity on T1-weighted images larger than 6 cm diameter, and peritumoral edema. The highest specificity is associated with tumor necrosis, tumor diameter larger than 8 cm and neurovascular involvement [10]. Another statistically significant aspect of malignancy included inhomogeneity on T2-weighted images and a change in pattern from homogeneity on T1-weighted images to inhomogeneity on T2-weighted images [11]. However, none of these elements alone can define a malignant tumor. This study proposed a method of predicting the malignancy of bone tumors from MRI scans by using pretrained residual network models and a fully connected neural network as a clinical model. With only one neuron as an output and Sigmoid as the activation function, the output of the clinical model is a real number between 0 and 1, representing the predicted malignancy for a set of images and clinical data provided as an input. For each ResNet classifier, accuracy, precision, recall and area under the curve were measured in order to assess the performance of each model. For the T1 classifier, a 93.67% accuracy was observed with a recall of 94.03%. The accuracy value is showing that the model is predicting correctly in both true positive and true negative cases while recall value indicates that the model correctly classifies the true positives. T2 classifier has slightly lower values for accuracy and recall due to missing T2 images in the MRI scans as described earlier. However, it is still performing well with 86.67% accuracy and 83.87% recall. Area under the curve for both classifiers is over 0.90 indicating that the models are not random classifiers, and the images are classified correctly both in training and validation stages. The clinical model, which was trained on the clinical data and the predicted values of T1 and T2 classifiers, has an accuracy of 80.84% and a recall of 94.38%. Since T1 classifier is performing better than T2 classifier due to more data available in the study, it can be seen that this has an effect on the clinical model in terms of precision. Withal, the ROC of the clinical model classifier shows a model which has the capacity of class separation. The proposed method was able to achieve good performance based on the dataset it was trained on but with noticeable difference in performance between the two ResNet classifiers. The difference in performance is due to the image availability for T1 versus T2. In the dataset, 1463 images were available for T1 compared with 664 images for T2. While data augmentation techniques were applied for both T1 and T2 images, there is still an imbalanced dataset which has an impact on the performance of the final model. Figure 7a,b represents a benign tumor from a 19 year old female patient in both T1 and T2 weighted images. The lesion is a tibia tumor located in the epiphysis part of the bone. For this image, the T1 classifier predicted the tumor as 0.02% of being malign, showing the fact that T1 classifier has the capacity to separate the classes correctly. 

**Figure 8:**
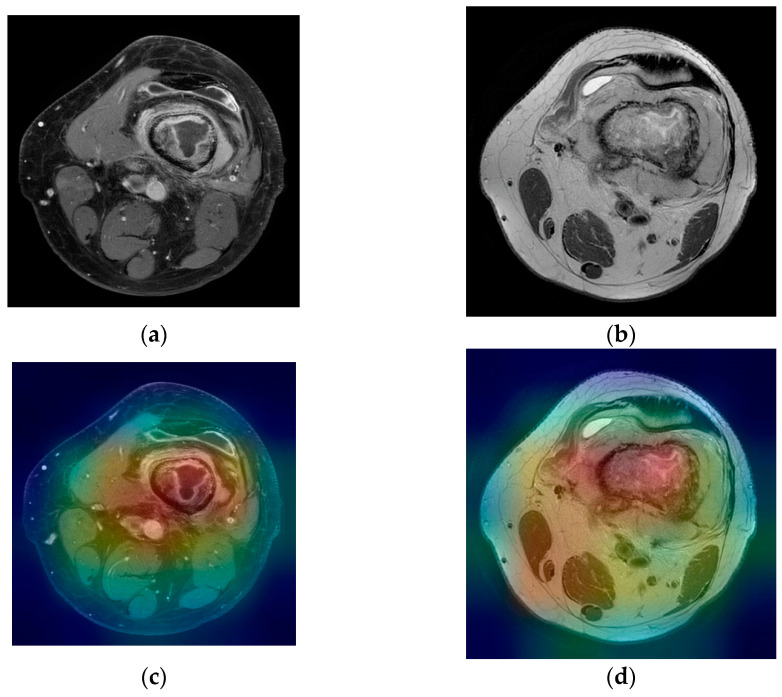
(**a**) Femur malignant tumor—T1 FS with contrast weighted image; (**b**) femur malignant tumor—T2 weighted image; (**c**,**d**) corresponding class activation maps.

For the T2 image, the corresponding T2 classifier predicted the tumor as 41.05% of being malign. While this prediction is still under the 50% threshold, it is showing the fact that the T2 ResNet classifier can introduce false positive predictions in the results. For each image presented in Figure 7a,b and Figure 8a,b, class activation map (CAM) was applied. Figure 7c,d and Figure 8c,d containing the CAM for these images, show that the two trained ResNet50 models from the proposed method are extracting relevant features regarding the lesion from the MRI scan.

In orthopedics, there are a few studies which use deep learning algorithms for classifying bone lesions. Compared with the method proposed by Feyisope R. Eweje et al. [1] which requires the segmentation of the images before training, the proposed method requires no prior segmentation due to the fact that the classifier used for T1 and T2 images is a pretrained ResNet50, which classifies the entire image as benignant or malign. Even more so, the clinical model is a neural network classifier, with an input layer of six neurons, one hidden layer with three neurons, and one neuron as an output (Figure 5). Besides sex, age, and bone type, it includes as inputs the bone location and the predicted malignancy values from T1 and T2 classifiers. However, the study has a significant lower number of cases compared with [1]. From the total number of 23 patients in the study, 14 were women with ages between 16 and 74 years old. While the clinical model has a good performance with 80.56% accuracy in the validation stage, the class imbalance of the dataset is still an open challenge since bone tumors are not common compared with other types of tumors. The method proposed in this paper can be considered a pilot study as it was limited by the number of cases, containing only 39 MRI scans from 23 patients. While the proposed work requires no manual segmentation thus minimal intervention from an imagist, it needs to be further tested on a larger cohort of patients. All models were trained in cloud on Google Colab [34] with Nvidia Tesla K80 by using Tensorflow [35] with Keras version 1.2.0 and Python version 3.7.0.

## 7. Conclusions

The diagnosis of bone tumors is given based on three distinct elements: symptomatology, imaging, and histopathological examination. An essential element of the effectiveness of the treatment is that it should be initiated as soon as possible after the onset of symptoms. Unfortunately, the symptoms are nonspecific and are often poorly represented in the early stages of bone tumors. Even if it is the most important step of the diagnostic algorithm, the histopathological diagnosis is based on an invasive technique, and it is time consuming due to the limitations of the procedure itself. It is obvious that the method which can bring fast data to differentiate a benign tumor from a malignant one is imaging. The differential diagnosis of the two types of tumors in the initial stages and the probability diagnosis of a certain type of malignant bone tumor are most often made on MRI elements. Improving the percentage of correct diagnoses from these points of view can be carried out using AI algorithms, which is useful for radiologists as well as for orthopedic surgeons. This work provides a useful tool which can be used to predict the malignancy of a bone tumor from MRI using deep learning algorithms. Moreover, a software based on AI algorithms trained on MRI images which did not require manual segmentation from a specialist, was designed. Thus, the proposed method can be an important tool for strengthening the diagnosis given by the orthopedist. 

## Figures and Tables

**Table 1:** Dataset detailed description.

Patient Number	No. of MRI Scans	Age	Gender	Bone	Location	Tumor Type
1	4	19	F	Tibia	Epiphysis	Benign (giant cell tumor)
2	1	25	F	Tibia	Epiphysis	Benign (giant cell tumor)
3	1	32	F	Femur	Diaphysis	Benign (non-ossifying fibroma)
4	3	15	F	Femur	Diaphysis	Benign (non-ossifying fibroma, enchondroma)
5	1	65	F	Femur	Diaphysis	Benign (enchondroma)
6	1	65	F	Fibula	Epiphysis	Benign (enchondroma)
7	1	66	F	Humerus	Diaphysis	Benign (enchondroma)
8	1	66	F	Phalange	Diaphysis	Benign (enchondroma)
9	2	35	M	Tibia	Diaphysis	Benign (enchondroma)
10	4	42	M	Tibia	Diaphysis	Benign (bone lipoma, enchondroma)
11	1	18	M	Femur	Diaphysis	Malignant (Ewing sarcoma)
12	1	45	M	Humerus	Epiphysis	Malignant (chondrosarcoma)
13	3	70	M	Scapula	Not applicable (N/A), wide bone	Malignant (chondrosarcoma)
14	2	54	F	Ilium	N/A, wide bone	Malignant (chondrosarcoma)
15	1	52	F	Scapula	N/A, wide bone	Malignant (osteorarcoma)
16	1	65	M	Femur	Epiphysis Diaphysis	Malignant (osteorarcoma)
17	1	40	F	Fibula	Epiphysis	Malignant (osteorarcoma)
18	2	68	M	Femur	Epiphysis	Malignant (renal carcinoma metastasis)
19	1	74	F	Ilium	N/A, wide bone	Malignant (renal carcinoma metastasis)
20	2	17	F	Humerus	Diaphysis	Malignant (osteosarcoma)
21	1	80	M	Sacrum	N/A, wide bone	Malignant (lung cancer metastasis)
22	1	35	M	Femur	Diaphysis	Malignant (scapular ostesarcoma metastasis)
23	3	16	F	Femur	Diaphysis	Malignant (osteosarcoma)

**Table 2:** ResNet50 architecture of the proposed method, adapted from K. He et al. [24].

Layer Stack Id	ResNet50
1	Convolution 7 × 7, 64 stride2 3 × 3 max pool, stride 2
	Output: 112 × 112
2	$3\times \left[\begin{array}{c} 1\times 1,\;64\\ 3\times 3,\;64\\ 1\times 1,\;256 \end{array}\right]$ Convolution
	Output: 56 × 56
3	$4\times \left[\begin{array}{c} 1\times 1,\;128\\ 3\times 3,\;128\\ 1\times 1,\;512 \end{array}\right]$ Convolution
	Output: 28 × 28
4	$6\times \left[\begin{array}{c} 1\times 1,\;256\\ 3\times 3,\;256\\ 1\times 1,\;1024 \end{array}\right]$ Convolution
	Output: 14 × 14
5	$3\times \left[\begin{array}{c} 1\times 1,\;512\\ 3\times 3,\;512\\ 1\times 1,\;2048 \end{array}\right]$ Convolution
	Output: 7 × 7
FC	Dropout, rate 0.3
	Flatten
	Dropout, rate 0.5
	1-d, Sigmoid activation function

**Table 3:** Encoded values for the clinical data.

Dataset Column	Value	Encoded Value
Gender	M	0
	F	1
Bone	Tibia	1
	Femur	2
	Humerus	3
	Phalanges	4
	Scapula	5
	Ilium	6
	Sacrum	7
	Fibula	8
Location	Wide bone	0
	Diaphysis	1
	Epiphysis	2

**Table 4:** Performance metrics of the image classifiers.

**Train Step**	**Accuracy**	**Recall**	**Precision**	**Area under the Curve**
T1 classifier	93.67%	94.03%	96.43%	0.9748
T2 classifier	86.67%	83.87%	89.66%	0.9071
**Validation Step**	**Accuracy**	**Recall**	**Precision**	**Area under the Curve**
T1 classifier	95.00%	95.52%	96.97%	0.9923
T2 classifier	95.00%	100%	85.71%	1.000

**Table 5:** Performance metrics of the clinical model.

	Accuracy	Recall	Precision	Area under the Curve
Train Step	80.84%	94.38%	75.68%	0.8845
Validation Step	80.56%	91.89%	75.56%	0.8788

## Data Availability

Anonymized data from the study can be obtained from the authors upon request.
